# *OsOFP3* Negatively Regulates Heat Stress Tolerance by Modulating H_2_O_2_ Homeostasis and Stomatal Aperture in Rice

**DOI:** 10.3390/antiox15030314

**Published:** 2026-03-02

**Authors:** Guiyuan Yu, Yingfeng Wang, Guilian Zhang, Huabing Deng, Wenbang Tang, Lifeng Wang, Yunhua Xiao

**Affiliations:** 1 College of Agronomy, Hunan Agricultural University, Changsha 410128, China; gyyu0325@hunaas.cn (G.Y.); wangyf@stu.hunau.edu.cn (Y.W.);; 2 Hunan Hybrid Rice Research Center, Changsha 410125, China; 3 Yuelushan Laboratory, Changsha 410128, China

**Keywords:** rice, heat stress, *OsOFP3*, *OsHTAS*, reactive oxygen species, stomata

## Abstract

Rice (*Oryza sativa*) is a staple crop that is highly susceptible to heat stress (HS), which severely impairs growth and yield. In this study, we identified the rice Ovate Family Protein *OsOFP3* as a novel negative regulator in response to heat. Our results demonstrate that the expression of *OsOFP3* is suppressed at both the transcriptional and protein levels under HS. Overexpression of *OsOFP3* significantly reduces the survival rate of rice seedlings under HS and exacerbates chlorophyll degradation, membrane damage, and the accumulation of reactive oxygen species (H_2_O_2_ and O_2_^−^). In contrast, *OsOFP3* mutants exhibit enhanced heat tolerance. Moreover, *OsOFP3*-overexpressing plants display increased stomatal opening and decreased stomatal closure under HS. Molecular interaction analysis further reveals that OsOFP3 interacts with the C-terminal domain of OsHTAS, a known positive regulator of heat tolerance encoding an E3 ubiquitin ligase, and this interaction depends on the RING domain of OsHTAS. Taken together, our findings indicate that *OsOFP3* negatively regulates rice heat tolerance by disrupting ROS homeostasis, inhibiting stomatal closure, and potentially antagonizing the OsHTAS-mediated signaling pathway. This research provides new insights into the molecular mechanisms underlying HS tolerance in rice.

## 1. Introduction

Under the exacerbation of global climate change, HS has become one of the major abiotic stresses limiting crop yield and quality [1]. Rice is the staple food for more than half of the world’s population [2]. The stability of rice production is crucial to food security. HS affect multiple critical growth stages of rice, directly disrupting the physiological metabolic balance. For photosynthesis, heat damages the structure and function of the thylakoid membrane and inhibits the activity of Rubisco activase (RCA), resulting in the decreased efficiency of photosystem II and carbon assimilation [3,4]. Scafaro also suggested that overexpression of RCA in wild rice significantly ensured the normal growth and increased the yield of cultivated rice under HS, suggesting that engineering RCA may be an effective way for heat tolerance breeding [5]. Studies have shown that when rice is exposed to HS, reactive oxygen species (ROS) accumulate within the cells, intensifying lipid peroxidation and protein oxidation of the cell membranes, thereby damaging the structure and function of the biological membranes and ultimately leading to cell death [6], increased chalky grain rate [7], seedling death [8], and even spikelet sterility [9,10]. In addition, the content of endogenous hormones will change when rice is subjected to stress. Research has demonstrated that under HS, the ethylene-mediated signaling pathway mitigates oxidative damage in rice seedlings, preserves chlorophyll content, and enhances plant heat stress tolerance [11]. HS can also induce an increase in the abscisic acid (ABA) content in rice grains and pollen, which leads to pollen abortion and yield reduction [12].

When plants are subjected to HS, they will initiate a series of complex physiological and molecular response mechanisms. The movement of stomata is the core link in the regulation of leaf water transpiration and body temperature; stomatal closing can alleviate water loss caused by HS, which is an important strategy for plants to improve heat tolerance [13]. Hydrogen peroxide (H_2_O_2_), as a kind of stable ROS, plays a dual role in this process: at low concentrations, it acts as a signaling molecule, participates in acclimatory signaling, and triggers tolerance to various biotic and abiotic stresses; and at high concentrations, it causes programmed cell death [14]. In stomatal guard cells, H_2_O_2_ is a key secondary messenger in the ABA signaling pathway, which can activate calcium channels and eventually induce stomatal closure [15]. Interestingly, an ABA-independent mechanism of stomatal closure was also revealed in rice: the drought and salt tolerance factor DST, functioning as a zinc finger transcription factor, negatively regulates H_2_O_2_-induced stomatal closure by directly modulating the expression of genes involved in hydrogen peroxide scavenging [16]. Therefore, maintaining cellular H_2_O_2_ homeostasis is a critical determinant of heat tolerance in rice.

The regulation of H_2_O_2_ homeostasis and stomatal movement relies on a sophisticated signaling network. The ubiquitin-26S proteasome system (UPS)-mediated post-translational modification is a core mechanism for plants to rapidly respond to environmental stress and precisely regulate signaling pathways [17]. The E3 ubiquitin ligase-containing RING domains play a critical “executor” role in this process by specifically recognizing and ubiquitinating substrate proteins [18]. Numerous studies have indicated that RING finger E3 ligases are extensively involved in the post-translational regulatory networks of plant hormone signaling and abiotic stress responses [19]. For instance, the RING E3 ligase XERICO positively regulates drought resistance by promoting ABA biosynthesis in *Arabidopsis thaliana* [20], while *HOS1* participates in low-temperature responses by negatively regulating cold signal transduction [21]. The C3HC4 RING finger E3 ligase OsDIS1 negatively regulates drought stress tolerance by modulating multiple stress-related genes and potentially through the post-translational modification of *OsNek6* in rice [22]. Hot pepper RING MEMBRANE-ANCHOR 1 HOMOLOG1 functions as an E3 ligase, ubiquitinating the plasma membrane aquaporins PIP2;1 under water-deficient conditions [23]. It is worth noting that the RING finger E3 ligase OsHTAS, as a positive regulator of heat tolerance, enhances the heat tolerance of rice seedlings by promoting H_2_O_2_ accumulation and inducing ABA-dependent stomatal closure [24].

The Ovate Family Proteins (OFPs) are a class of plant-specific transcription factors that are extensively involved in various processes such as plant growth and development and hormone signal transduction [25]. The OFP family was originally identified in tomato as a key quantitative trait locus controlling fruit shape; overexpression of *OFP* leads to phenotypic changes such as plant dwarfing and organ size reduction [26]. A total of 19 *OFP* gene family members have been identified in *Arabidopsis thaliana*, 33 in rice, and 45 in maize (*Zea mays*) [27]. Overexpression of *OsOFP2* resulted in plant dwarfing and leaf morphological changes [28]. Overexpression of *OsOFP3* resulted in BR insensitivity and decreased plant height, while deletion of *OsOFP3* promoted seedling growth [29]. *OsOFP6* is expressed in various tissues of rice, and *OsOFP6*-RNAi plants exhibited faster water loss and higher H_2_O_2_ contents under drought conditions [30]. *OsOFP8* plays a positive role in BR signaling [31]. Overexpression of *OsOFP19* leads to reduced plant height, thicker leaves, and sturdier stems in rice [32]. These studies revealed the multiple functions of the *OFP* family in plant growth and development. Although a few *OFP* members have been confirmed to participate in abiotic stress responses, their specific functions and molecular mechanisms in heat stress response remain poorly understood.

In this study, we identified a rice gene, *OsOFP3,* that was significantly inhibited by HS. Analysis revealed that overexpression of *OsOFP3* aggravated chlorophyll degradation, membrane damage, and reactive oxygen species accumulation under HS. Furthermore, OsOFP3 interacts with OsHTAS, which may negatively regulate heat tolerance by modulating the H_2_O_2_ signaling pathway.

## 2. Materials and Methods

### 2.1. Plant Materials and High-Temperature Stress Treatments

Zhonghua 11 (*Oryza sativa* L. subsp. *japonica* cv. ZH11) was used as a control, and the overexpressed lines (*3o-1*, *3o-2*) and mutant lines (*ofp3-1*, *ofp3-2*) were constructed on ZH11 as the genetic background. We produced OsOFP3 knockout mutants using CRISPR/Cas9 editing. By targeting a sequence at the 50-end of the coding sequence, some independent homozygous mutant lines were obtained. Two alleles, *ofp3-1* and *ofp3-2*, containing a 1 bp insertion and a 386 bp deletion, respectively, were selected for the detailed analysis. The OsOFP3-Flag fusion overexpressed material was used to examine protein expression under HS. The seeds were immersed at 37 °C to accelerate germination (changing water for 12 h). The well-germinated seeds were seeded in a 96-well PCR plate with holes in the bottom, and then hydroponic culture was carried out in a light incubator at 28 °C. The culture was changed to modified Kimura B nutrient solution from the fourth day of seeding, and the nutrient solution was replaced on days 4, 6, 8, 9, and 10. The light incubator was set to a light cycle of 12 h light/12 h dark, light intensity of 540 μmol m^−2^ s^−1^, relative humidity of 70%, normal temperature treatment of 28 °C, and heat treatment of 42 °C or 45 °C. The materials could not survive for a long time at 45 °C. In order to observe the change trends of the plant morphology and physiological indexes under the high-temperature treatment for 5 days, the temperature was changed to 42 °C.

### 2.2. Expression Analysis of OsOFP3 Under High-Temperature Stress

The wild-type ZH11 was used as the material, and the seedlings were treated at 45 °C for 0, 2, 4, and 8 h. The extraction of total RNA, reverse transcription, and qRT-PCR detection were performed according to the method described previously [33]. The gene expression was quantified using the 2^−∆∆CT^ method, and three biological replicates were applied throughout the assay. The primer sequences used for qRT-PCR are shown in Supplemental Table S1. The *OsOFP3*-Flag fusion overexpressed material was used for Western blotting. After sampling at the same time points, the protein was extracted, separated by SDS-PAGE, wet transferred to the membrane, incubated with antibody, and protein expression changes were ultimately analyzed by ECL chemiluminescence [34].

### 2.3. NBT and DAB Staining

NBT and DAB staining were performed according to the method described by Kaur [35]. The specific steps were as follows: Rice leaves treated at 42 °C for 72 h were cut into approximately 3 cm segments and separately immersed in DAB solution (1 mg/mL) and NBT solution (1 mg/mL) prepared with double-distilled water (pH 3.8) and were penetrated under vacuum at 60 kpa for 10 min. The samples were subsequently immersed in absolute ethanol and then stored in a water bath at 100 °C until the chlorophyll faded completely. Finally, images were captured using a microscope.

### 2.4. Chlorophyll and Electrolyte Leakage Detection

Leaf samples of 0.1 g were weighed and cut into pieces, 95% acetone-ethanol extract was added, and the samples were wrapped in tin foil and placed in the refrigerator at 4 °C overnight until the filaments turned white. The absorbance values were measured at wavelengths of 645 nm and 663 nm. The chlorophyll concentration was calculated according to the formulas, Ca = 12.7 × A_663_ − 2.69 × A_645_, Cb = 22.9 × A_645_ − 4.86 × A_663_, Total chlorophyll concentration = Ca + Cb. A_645_ is the absorbance value of the extract at 645 nm, A_663_ is the absorbance value of the extract at 663 nm. Chlorophyll content = CV/(1000A), where C represents the chlorophyll concentration (mg·L^−1^), V represents the total volume of extract (mL), and A represents the fresh weight of leaves (g).

The leaves were washed with distilled water, blotted to dry the surface water, and cut into about 4 cm long strips avoiding the main vein. Next, 0.1 g of sample was placed in 10 mL of distilled water for 12 h before the conductance value (R_1_) was determined. Then, the samples were heated in a water bath at 100 °C for 30 min, cooled to room temperature, shaken, and the conductance value (R_2_) was measured again. The relative electrical conductivity was calculated as R_1_/R_2_ × 100%. The conductivity meter was calibrated at two points with a standard solution before use.

### 2.5. Measurement of the Degree of Stomatal Opening

Seedlings were sampled after 2 h of heat treatment at 45 °C. To reduce the influence of position, individuals with uniform growth that were located in the middle region of the culture apparatus were selected, and the use of edge row materials was avoided. About 3–5 mm of tissue was taken from the shoot tip, and three biological replicates were set for each material. Immediately after sampling, samples were transferred to 2 mL EP tubes containing electron microscope fixative, evacuated for 5 h, and subsequently stored wrapped in tin foil at 4 °C until examination. After scanning electron microscope observation, three pictures of each treatment group (room temperature/high temperature) were selected under the same field of view, and 50 stomata were counted in each picture. The stomata were classified and counted according to their opening state (completely open, partly open, completely closed).

### 2.6. Yeast Two-Hybrid Assays

The coding sequences of the C-terminus containing the RING finger domain (amino acids 338–414), the N-terminus (amino acids 1–337), and the full-length OsHTAS protein were amplified and cloned in frame with the GAL4 DNA binding domain of the pGBKT7 vector to generate GAL4 DNA-BD fusion constructs, called BD-OsHTAS(C), BD-OsHTAS(N) and BD-OsHTAS, respectively. Then, the coding sequence of *OsOFP3* was amplified and cloned in pGADT7, called AD-OsOFP3. The prey and bait proteins were co-transformed into AH109 yeast cells using the lithium acetate method, and transformants were selected on synthetic dextrose/–Ade/–His/–Leu/–Trp plates. Colony growth was observed after 3–5 days of incubation at 30 °C. AD-OsOFP3 + BD-GSK229 was used as a positive control, and the empty vector combination was used as a negative control.

### 2.7. Statistical Analysis

All results are presented as the mean ± SD (n = 3). Statistical analyses were performed using DPS (v7.05). One-way ANOVA was used to statistically analyze the data, and differences were considered significant at *p* < 0.05 by Duncan’s multiple range test.

## 3. Results

### 3.1. OsOFP3 Expression and Protein Accumulation Decreased Under HS

**Figure 1:**
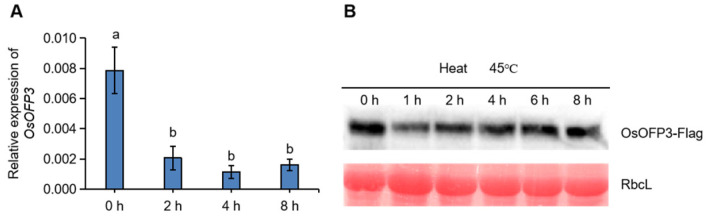
The transcriptional and protein levels of OsOFP3 under heat stress. (**A**) Relative expression of *OsOFP3* under heat stress. (**B**) The OsOFP3 protein accumulation under heat stress was detected by Western blotting. Data are means ± SD. Different lowercase letters show significant differences among treatments analyzed by one-way ANOVA comparison test (n = 3, *p* < 0.05).

We used Plant CARE (http://bioinformatics.psb.ugent.be/, accessed on 15 March 2025) to analyze the cis-acting elements in the *OsOFP3* promoter region, which is rich in multiple response elements related to plant hormones, such as ABA and Gibberellic acid (GA), as well as to anaerobic and low temperature stress (Supplemental Figure S1). The result implies that *OsOFP3* may be involved in plant growth and development and stress resistance. To explore the possible regulatory mechanism of *OsOFP3* in HS, we analyzed the changes in *OsOFP3* at the transcriptional and protein levels by using quantitative real-time PCR and Western blotting, respectively. The results showed that the expression level of *OsOFP3* significantly decreased over time in rice plants treated at 45 °C (Figure 1A). Western blot analysis showed that protein accumulation significantly decreased after one hour of heat treatment, followed by a gradual recovery, although it remained lower than the control (Figure 1B). These results indicate that HS suppresses OsOFP3 accumulation at both the transcriptional and protein levels, suggesting that *OsOFP3* may participate in the heat response by down-regulating the expression and protein degradation.

### 3.2. OsOFP3 Negatively Regulates Heat Tolerance During the Seedling Stage in Rice

**Figure 2:**
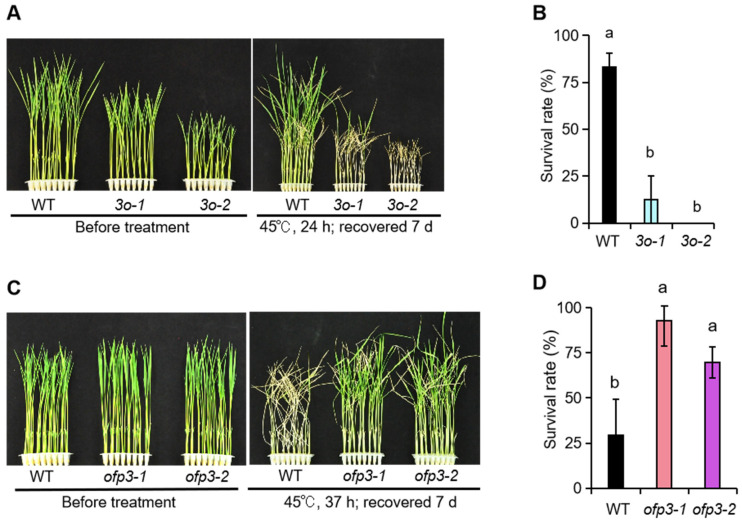
*OsOFP3* negatively regulates heat tolerance in rice seedlings. (**A**) Representative phenotypes of wild-type (WT), *3o-1*, and *3o-2* before and after 24 h of heat stress treatment (45 °C). (**B**) Survival rate of *OsOFP3*-overexpressing plants. (**C**) Representative phenotypes of wild-type (WT), *ofp3-1* and *ofp3-2* before and after 37 h of heat stress treatment (45 °C). (**D**) Survival rate of *OsOFP3* mutant plants. Data are means ± SD. Different lowercase letters show significant differences among treatments analyzed by one-way ANOVA comparison test (n = 3, *p* < 0.05).

To further investigate the effect of *OsOFP3* on rice under HS, we subjected WT, *OsOFP3*-overexpressing plants (*3o-1*, *3o-2)*, and *OsOFP3* mutants (*ofp3-1*, *ofp3-2)* to high-temperature treatment. The results showed that the survival rate of both *3o-1* and *3o-2* was significantly lower than that of the WT. The survival rate of the WT was more than 80%, while that of *3o-1* was less than 25%, and all the *3o-2* plants died (Figure 2A,B). The survival rates of both *ofp3-1* and *ofp3-2* were significantly higher than the WT (Figure 2C,D). These results suggest that *OsOFP3* negatively regulates the tolerance of rice seedlings to HS.

### 3.3. OsOFP3 Overexpression Leads to Accelerated Chlorophyll Decomposition, More ROS, and Enhanced Ion Efflux Under HS

**Figure 3:**
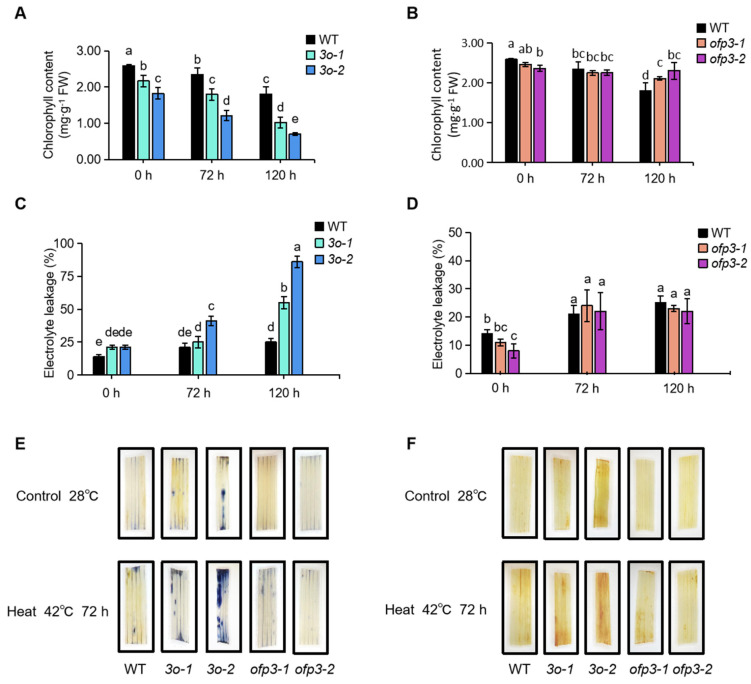
Exacerbation of heat-induced physiological damage by *OsOFP3* overexpression. (**A**) Chlorophyll content determination of *OsOFP3*-overexpressing plants. (**B**) Chlorophyll content determination of *OsOFP3* mutant plants. (**C**) Relative electrolyte leakage of *OsOFP3*-overexpressing plants. (**D**) Relative electrolyte leakage of *OsOFP3* mutant plants. (**E**) Histochemical staining of NBT. (**F**) Histochemical staining of DAB. Data are means ± SD. Different lowercase letters show significant differences among treatments analyzed by one-way ANOVA comparison test (n = 3, *p* < 0.05).

To understand the physiological mechanism of *OsOFP3* under HS, the chlorophyll content was measured after 72 h and 120 h. The results showed that the chlorophyll content of all plants decreased with the extension of high-temperature treatment time. After 72 h of treatment, the chlorophyll content of *3o-1* and *3o-2* was significantly lower than WT, while there was no significant difference between the knockout plants and the WT. After 120 h of treatment, the chlorophyll content of *ofp3-2* was significantly higher than WT, while *3o-2* was less than half of the wild type (Figure 3A,B). This suggests that the overexpression of *OsOFP3* accelerates chlorophyll decomposition under HS.

Under the same treatment conditions, the electrolyte leakage of *3o-2* was significantly higher than the WT at 72 h of heat treatment, but there was no significant difference in the knockout plants. After 120 h of heat treatment, the electrolyte leakages of *3o-1* and *3o-2* reached 50% and 80%, respectively, which were significantly higher than the WT, while the electrolyte leakages of *ofp3-1* and *ofp3-2* were slightly lower than the WT (Figure 3C,D). This result indicates that the cell membrane of the overexpressing plants was more severely damaged under HS, resulting in more ion efflux.

The accumulation of H_2_O_2_ and O_2_^−^ in leaves was detected by DAB and NBT staining after 72 h of heat treatment. The results showed that all the plant leaves exhibited increased brown and blue spots. However, the spots in the overexpressing plants were significantly darker than the WT, while the knockout plants showed a staining intensity similar to the WT (Figure 3E,F). This indicates that HS induces higher levels of ROS in overexpressing plants, potentially leading to more severe damage to the cell membrane system.

### 3.4. The Stomatal Aperture of the OsOFP3-Overexpressing Plants Was Higher

**Figure 4:**
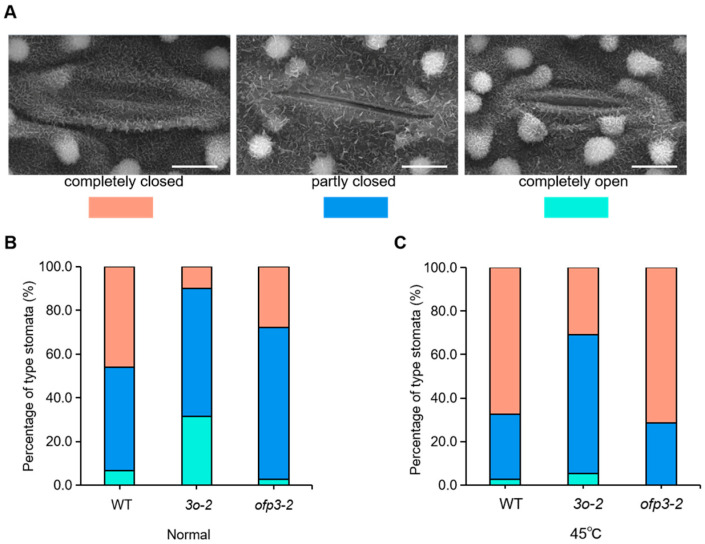
Stomatal aperture under control and heat stress conditions. (**A**) Scanning electron microscopy images of three levels of stomatal opening. Percentages of three levels of stomatal opening in cv ZH11 and *OsOFP3* mutant plants under normal conditions. From left to right: fully closed, partially open, and fully open stomata. Bars = 5 µm. (**B**) Percentages of three levels of stomatal opening in WT, *3o-2* and *ofp3-2* under normal conditions. (**C**) Percentages of three levels of stomatal opening in WT, *3o-2* and *ofp3-2* under heat stress conditions (45 °C for 2 h).

To investigate whether *OsOFP3* is involved in this process, WT, *3o-2* and *ofp3-2* were treated at 45 °C for 2 h, followed by scanning electron microscopy. The results showed that the number of stomata that were opened at 45 °C was lower than that at room temperature. However, the stomatal opening rate of *3o-2* at 45 °C was 69.3%, which was about twice that of the WT (34.6%), while the opening rate of *ofp3-2* was only 28.7%, which was slightly lower than the WT (Figure 4). This suggests that *OsOFP3* may regulate rice heat tolerance by affecting stomatal opening and closing. Excessive stomatal opening in overexpressing plants under HS may lead to increased water loss, thereby reducing heat tolerance.

### 3.5. Interaction Between OsOFP3 and OsHTAS in Yeast

**Figure 5:**
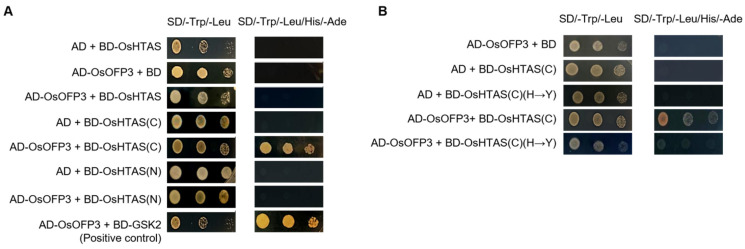
Yeast two-hybrid analysis of the interaction between OsOFP3 and OsHTAS. (**A**) Identification of the interaction between OsOFP3 and the N-terminus, C-terminus, or full-length OsHTAS. AD-OsOFP3 and BD-GSK2 were used as positive controls. (**B**) Identification of the interaction between OsOFP3 and the RING finger domain of OsHTAS. H→Y indicates that the 358th amino acid of OsHTAS was changed from His to Tyr.

Previous studies have shown that the RING finger E3 ligase OsHTAS enhanced heat tolerance by promoting H_2_O_2_ accumulation-induced stomatal closing in rice [24]. To explore whether there is an interaction between OsOFP3 and OsHTAS, we conducted a yeast two-hybrid experiment. The N-terminus of OsHTAS contains four transmembrane (TM) domains, and the C-terminus contains a RING finger domain. We constructed vectors by fusing the full-length OsHTAS (HTAS, amino acids 1–414), N-terminal region (OsHTAS(N), amino acids 1–337), or C-terminal region (OsHTAS(C), amino acids 338–414) to the BD domain. The results showed that OsOFP3 did not interact with HTAS or OsHTAS(N) but has positive interactions with OsHTAS(C) (Figure 5A). To test whether the intact RING finger domain (amino acids 338–378) of OsHTAS was indispensable for the interaction between OsHTAS(C) and OsOFP3, We introduced a point mutation by substituting histidine (His-358) with tyrosine (Tyr-358), which disrupted the catalytic core of the RING finger domain. New vectors were reconstructed and denoted as BD-OsHTAS(C)(H→Y). The results showed that BD-OsHTAS(C)(H→Y) did not interact with OsOFP3 (Figure 5B), indicating that the RING finger domain is required for the interaction between OsHTAS(C) and OsOFP3.

## 4. Discussion

### 4.1. OsOFP3 Is Heat-Suppressive and Negatively Regulates Heat Tolerance in Rice

In this study, phenotypic analysis clearly confirmed that *OsOFP3* is a key factor that negatively regulates heat tolerance at the rice seedling stage. In contrast to OsHTAS, a positive regulator of heat tolerance, overexpression of *OsOFP3* significantly reduced plant survival under HS, whereas mutant plants showed greater heat tolerance. This “overexpression-sensitive, mutant-tolerant” phenotypic pattern clearly defines the negative regulatory function of *OsOFP3*. More importantly, we found that the expression of *OsOFP3* was suppressed by HS both at the transcriptional and protein levels, indicating that OsOFP3 is a heat-suppressive protein. The expression pattern is consistent with its negative regulatory function: when exposed to high temperature, the inhibition of heat stress tolerance was relieved by down-regulating OsOFP3 expression. This coordinated regulation at the transcriptional and protein levels is a common pattern found in other heat-reactive factors such as *HTS1* [36].

### 4.2. OsOFP3 Exacerbates Heat Damage by Disrupting Chloroplast Stability, ROS Homeostasis, and Stomatal Aperture

Overexpression of *OsOFP3* aggravated chlorophyll degradation, enhanced cell membrane damage (increased relative conductivity), and led to a large accumulation of reactive oxygen species (H_2_O_2_ and O_2_^−^), which are typical characteristics of plant oxidative damage under HS. First, a decrease in the chlorophyll content is typically linked to thylakoid damage and impaired photosynthetic function [3]. Overexpression of *OsOFP3* may have impaired chloroplast stability and disrupted chlorophyll metabolism under HS. Second, excessive reactive oxygen species (ROS) act as a central hub in various stress responses. This study confirmed that *OsOFP3*-overexpressing plants accumulated more H_2_O_2_ and O_2_^−^ under HS, which may be due to the inhibition of antioxidant enzyme activity or the promotion of enzymes that produce ROS, as observed in the heat-sensitive rice variety IR64 [37]. Excessive ROS attack biofilms, proteins, and nucleic acids, leading to membrane lipid peroxidation and ion leakage, ultimately accelerating cell death [38]. Third, stomata are critical for regulating transpiration, heat dissipation and water balance. We found that stomatal opening was greater in *OsOFP3*-overexpressing plants under HS, indicating that stomatal closure was inhibited, thereby destroying the water retention capacity, and may be a direct cause of their reduced heat tolerance. This phenotype is consistent with the functions of several genes that modulate stomatal closure by regulating H_2_O_2_ signaling, thereby altering heat tolerance. For instance, the positive regulators *OsHTAS* and *OsMDHAR4* promote heat tolerance by inducing stomatal closure through facilitating and mediating H_2_O_2_ signaling [39], respectively. In contrast, *OsOFP3* functions oppositely.

### 4.3. The Interaction Between OsOFP3 and OsHTAS May Reveal a Novel Regulatory Pathway for Heat Tolerance

The important finding of this study was the identification of OsHTAS as an interacting protein with OsOFP3 by the yeast two-hybrid assay. OsHTAS is a characterized E3 ligase that positively regulates rice heat tolerance by inducing stomatal closure through the promotion of H_2_O_2_ accumulation and the ABA signaling pathway. The identification of this interaction provides a key clue for unraveling the negative regulatory mechanism of *OsOFP3*. We hypothesized that *OsOFP3* may function as a negative regulatory component or antagonist in the *OsHTAS* signaling pathway. The specific mechanism may include the following hypothesis: OsHTAS may target OsOFP3 through ubiquitination modification and direct its degradation through the 26S proteasome. The present study found a rapid decrease in OsOFP3 protein levels under HS, a process that is most likely mediated by OsHTAS. The degradation of OsOFP3 is a key step in relieving heat resistance.

## 5. Conclusions

In this study, we systematically identified *OsOFP3* as a novel negative regulator of heat tolerance in rice. The expression of *OsOFP3* was inhibited by HS, and its overexpression significantly reduced plant heat tolerance, while the mutant showed enhanced heat tolerance. At the molecular physiological level, *OsOFP3* plays a negative regulatory role by aggravating chlorophyll degradation, promoting membrane system damage, enhancing ROS burst, and inhibiting stomatal closure under HS. Further mechanistic studies showed that OsOFP3 interacted with the E3 ligase OsHTAS, suggesting that *OsOFP3* may function by modulating OsHTAS-mediated H_2_O_2_ signaling and the stomatal closure pathway.

## Data Availability

The original contributions presented in this study are included in the article/Supplementary Material. Further inquiries can be directed to the corresponding author.

## Supplementary Materials

The following supporting information can be downloaded at: https://www.mdpi.com/article/10.3390/antiox15030314/s1, Table S1: The list of primer pairs; Figure S1: Analysis of promoter homeostasis elements of *OsOFP3.*

## Abbreviations

The following abbreviations are used in this manuscript:

HS	Heat stress
ROS	Reactive oxygen species
ABA	Abscisic acid
UPS	Ubiquitin-26S proteasome system
GA	Gibberellic acid
RCA	Rubisco activase
TM	Transmembrane domains
OFP	Ovate Family Protein
