# Supplementary material for: OsOFP3 Negatively Regulates Heat Stress Tolerance by Modulating H2O2 Homeostasis and Stomatal Aperture in Rice
Source: Antioxidants (Basel). 2026 Mar 2;15(3):314. doi: 10.3390/antiox15030314 (PMC13023431; doi:10.3390/antiox15030314)
Supplement: Supplementary file 1 [file antioxidants-15-00314-s001.zip › antioxidants-4162193-supplementary.pdf]

Supplementary Materials

Table S1. The list of primer pairs.

Primer name	Sequence (5'–3')
OsActin1-F	GACCTTCAACACCCCTGCTA
OsActin1-R	GAGTCCAACACAATACCTGTGG
OsOFP3-qRT-F	CGTCGGACTTCTACAACTGC
OsOFP3-qRT-R	TTGATTTGCGGAAGTCCTCG
AD-OsOFP3-F	CATCGATACGGGATCCATATGGACGGCGGCGGCTCA
AD-OsOFP3-R	TCATCTGCAGCTCGAGTCAGGGGAAAAGCGTCTC
BD-OsHTAS-F	CATGGAGGCCGAATTCATGGAGCATGCTACCTGTG
BD-OsHTAS-R	GCAGGTCGACGGATCCCTATACAGTGAATCTCGAG
BD-OsHTAS(C)-F	CATGGAGGCCGAATTCTGCTGCATCTGTCTTACTA
BD-OsHTAS(C)-R	GCAGGTCGACGGATCCCTATACAGTGAATCTCGAG
BD-OsHTAS(N)-F	CATGGAGGCCGAATTCATGGAGCATGCTACCTGTG
BD-OsHTAS(N)-R	GCAGGTCGACGGATCCTTACACAGCATCTTCAGCTGAA
BD-OsHTAS(C)(H→Y)-F	CATGGAGGCCGAATTCAGCTTCCTTGCACCTACTTCTTTCATGTGCA
BD-OsHTAS(C)(H→Y)-R	GCAGGTCGACGGATCCTGCACATGAAAGAAGTAGGTGCAAGGAAGCT

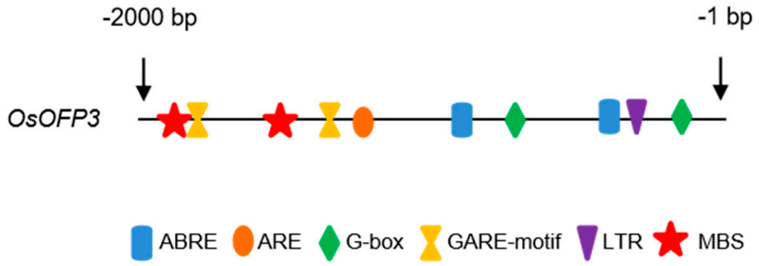

Figure S1. Analysis of cis-acting elements in the promoter of *OsOFP3*

A: ABRE (abscisic acid-responsive element), B: ARE (anaerobic induction regulatory element), C: G-box (light responsive element), D: GARE (gibberellin-responsive element), E: LTR (low temperature response element), F: drought-responsive element (MBS, MYB binding site).
